# Stability of Gastric Fluid and Fecal Microbial Populations in Healthy Horses under Pasture and Stable Conditions

**DOI:** 10.3390/ani14202979

**Published:** 2024-10-16

**Authors:** Rebecca C. Bishop, Ann M. Kemper, Lindsay V. Clark, Pamela A. Wilkins, Annette M. McCoy

**Affiliations:** 1 Department of Veterinary Clinical Medicine, University of Illinois Urbana-Champaign, Urbana, IL 61802, USA; 2 High-Performance Computing in Biology, Roy J. Carver Biotechnology Center, University of Illinois Urbana-Champaign, Urbana, IL 61802, USA

**Keywords:** microbial profiling, equine, gastrointestinal, colic

## Abstract

Equine gastrointestinal microbial communities vary across the gastrointestinal tract and in response to diet or disease. Understanding the composition and stability of gastric fluid microbiota in healthy horses is a prerequisite to understanding changes associated with the development of disease. The objective of this study was to describe microbial communities in the gastric fluid and feces of healthy horses longitudinally. Horses were maintained on pasture (6 weeks), stabled (5 weeks), then returned to pasture. A consistent forage diet was provided throughout. Native gastric fluid and feces were collected weekly for full-length 16S ribosomal DNA sequencing and microbial profiling analysis. Fewer taxa were identified in the gastric fluid (770) than in the feces (5284). Species richness and diversity were significantly different between sample types (*p* < 0.001), but not between housing locations (*p* = 0.3). There was a significant effect of housing and horse on the Bray–Curtis compositional diversity of gastric (*p* = 0.005; *p* = 0.009) and fecal (*p* = 0.001; *p* = 0.001) microbiota. When horses moved from pasture to stable, the relative proportions of gastric fluid Lactobacillaceae increased and Streptococcaceae decreased, while fecal Firmicutes increased and Bacteriodota decreased. Within each housing condition, there was no significant week-to-week variation in gastric (*p* = 0.9) or fecal (*p* = 0.09) microbiota. Overall, these findings support the maintenance of stable gastric and fecal microbial populations under each management condition, providing a basis for further investigation of gastric fluid microbiota in diseases of the foregut.

## Simple Summary

Intestinal microbiota (the gut microbiome) are important for normal gut function, especially in horses which rely on gut bacteria to break down plant material in the hindgut. Understanding normal microbiota is essential to be able to assess changes that occur during disease or in response to treatments. Post-mortem studies and biopsies have found different populations in the stomach mucosa (lining) compared to feces, but the gastric fluid has not been evaluated. The objective of this study was to describe the gastric fluid microbiome of healthy horses over time, under two housing conditions, and to compare the gastric fluid to fecal microbiome of paired samples. We found that while there were fewer bacteria (taxa) identified in the gastric juice compared to feces, there was a stable population of gastric microbiota which did not vary from week-to-week under either housing condition. There was a significant difference in compositional diversity (the relatedness of taxa present) between housing conditions, with changes in the relative proportions of a few key groups when horses moved from pasture to stable. These findings are important to inform future investigations of the gastric fluid microbiota in horses.

## 1. Introduction

Intestinal microbiota have long been recognized for their role in the digestion and breakdown of complex food particles and their role in the development of disease states is of increasing interest. Horses are exceptionally susceptible to gastrointestinal disturbances, often presenting as clinical signs of colic. Colic has a significant impact on equine industries and is a major cause of death in adult horses [1]. Intensive management of modern equines, including increased high energy concentrate feeds and decreased dietary fiber, increase the risk of gastrointestinal disturbance and disrupt the normal microbial homeostasis [2,3,4,5,6,7,8]. Abrupt diet change may lead to colic in horses, which is an association likely mediated by alterations in diet-adapted microbial populations [9].

In previous studies, the equine fecal microbiome showed > 65% stability over 6 weeks with consistent diet and management [10]. Core components of the equine fecal and large intestine microbiota have been reported, but this core community appears to be made up of a large number of low abundance microbiota, with marked variation between individuals [10,11,12,13,14,15]. Differences in fecal microbiota have been demonstrated in clinically normal horses in different geographic areas, under different management systems, and of different breeds or ages [10,13,16]. Dramatic dietary changes (i.e., high starch diets vs roughage only) are known to affect the fecal microbiome [4,6,8,17].

Regional anatomic and physiologic adaptations within the gastrointestinal tract [18,19,20] are reflected in the local microbial population. Distinct differences have been identified between the equine foregut (stomach and small intestine) and hindgut (cecum and colon) microbiota [11,12,20,21,22]. Recently, a unique microbial population was identified in the cecum that was separate from both small intestine and large colon [23]. Despite this knowledge, fecal samples are most commonly used to study the equine microbiome, and the equine gastric fluid microbiome largely remains a mystery, although it is reportedly significantly different from the well-characterized large intestinal and fecal microbiome [11,20]. To our knowledge, very little work has been conducted to characterize the microbiota of native gastric fluid in the horse. Additionally, the effect of subtle diet change (pasture- vs hay-based forage diets) on the equine microbiome has not been documented.

The objective of this study was to evaluate changes in the gastric fluid and fecal microbial populations over time when healthy horses were maintained on pasture, then stabled with forage diet, and subsequently returned to pasture. Gastric fluid was collected by a sterile double-tube technique without additional exogenous fluid. We hypothesized that the gastric fluid microbial community would be more diverse with fewer core bacterial families than the fecal microbiome. We further hypothesized that both gastric fluid and fecal microbial communities would remain stable over time under each management condition but would have significant differences in community membership and structure between pasture and stable management.

## 2. Materials and Methods

### 2.1. Study Population

Healthy adult horses were recruited from the teaching herd maintained at the University of Illinois Veterinary Teaching Hospital (VTH) in Urbana, Illinois. Inclusion criteria were adult horses (>3 years old) with no evidence of systemic disease, no history of antimicrobial administration, surgical procedure, or pregnancy in the preceding 3 months. Health status was determined based on physical examination, serum biochemistry profile and complete blood counts, as well as known health history on all horses for >1 year prior to study enrollment. Body condition score was determined at the time of study enrollment [24]. All study procedures were approved by the University of Illinois Institutional Animal Care and Use Committee (protocol #17140).

### 2.2. Husbandry

**Figure 1:**
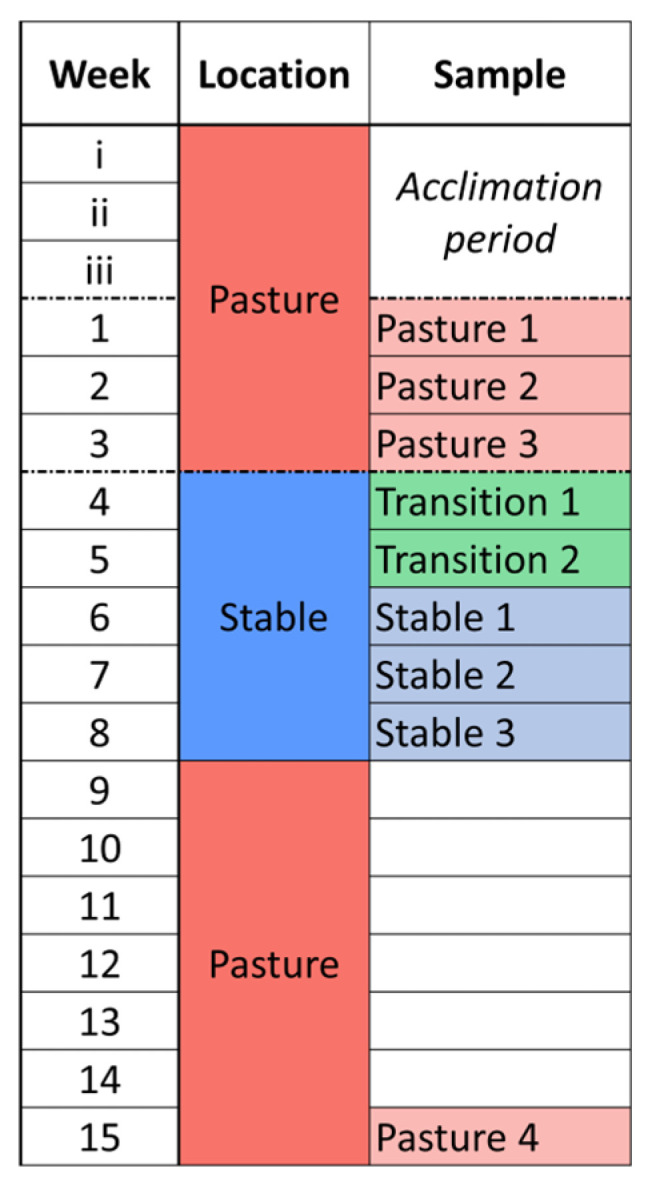
Study schedule showing horse housing location and sample collection timing.

The study period occurred from May–July 2020. At the time of enrollment, all horses had been with the VTH herd for >1 year and were accustomed to both group pasture housing and individual stall facilities. Horses were maintained on pasture in a single herd at the VTH for a minimum of 3 weeks prior to beginning the study to mitigate changes related to transportation from the Veterinary Medical Research Farm (1.8 miles). While on pasture, the horses had free access to water, grass, and grass/alfalfa mixed hay. After three weeks of sample collection, the horses were moved to individual indoor stalls within the VTH, where they had free access to water and grass/alfalfa mixed hay. Horses were hand-walked for 10 min daily during stabling period but were not allowed to graze. The first two weeks of indoor housing we labeled “Transition” and the following three weeks as “Stable” (Figure 1). Upon completion of the 5-week stabling period, horses were returned to pasture for 6 weeks, after which a final set of samples was collected. Horses were monitored daily by study personnel for attitude, appetite, and manure production throughout the study. Physical examinations were performed and horses were weighed weekly. Blood samples were collected by direct jugular venipuncture weekly for packed cell volume (PCV) and total solids (TS), as well as complete blood count (CBC) and fibrinogen to monitor for changes in systemic inflammatory state.

### 2.3. Sample Collection

Horses were restrained routinely (halter and lead rope, stocks, and nose twitch, if needed) with standing sedation using detomidine (0.01 mg/kg IV; Dormosedan™, Zoetis US, Parsippany, NJ, USA) or xylazine (0.5 mg/kg IV; Xylamed™, VetOne, Boise, ID, USA) as needed. A sterile nasogastric tube (Jorvet, Loveland, CO) with pre-placed sterile flexible inner tube (polyvinylchloride, 3/8” OD, 10 ft long, Everbilt, Atlanta, GA, USA) was passed through the nares, nasopharynx, esophagus, and into the stomach. Once in the stomach, the inner tube was advanced and gentle negative pressure was applied by 60 mL catheter tip syringe (Monoject™, Cardinal Health, Dublin OH, USA) until gastric contents were obtained. A minimum of 5 mL gastric fluid was collected in this manner without introduction of additional fluid into the stomach. Feces (minimum 2 g) were collected from the rectum manually by study personnel wearing a lubricated sterile sleeve. Samples were placed in sterile containers and stored on ice prior to freezing at −80 °C. A 0.5 mL aliquot of gastric fluid was used for measurement of pH using a portable electronic pH meter (Ohaus Corporation, Parsippany, NJ, USA).

### 2.4. Microbial DNA Preparation and Sequencing

Gastric fluid and fecal samples were thawed at room temperature and mixed prior to removing a 250 μg aliquot for microbial DNA isolation. Gastric fluid samples were centrifuged at 10,000× *g* for 10 min, the supernatant was separated, and 250 μg of the solids were weighed. If a sample had insufficient solid material, supernatant was added to a total weight of 250 μg. Samples were processed using the QIAamp PowerFecal Pro DNA Kit (Qiagen, Germantown, MD, USA) following the manufacturer’s protocol to isolate bacterial DNA. The eluted DNA concentration was quantified by fluorometry (Qubit 1x dsDNA HS kit, Invitrogen, Carlsbad, CA, USA) and the samples were stored at −80 °C until library preparation.

Library construction and sequencing using PacBio Sequel II were performed at the Roy J. Carver Biotechnology Center, University of Illinois at Urbana-Champaign. Amplicons were amplified using the Shoreline Complete ID kit (Shoreline Biome, Farmington, CT, USA), which amplifies a 2500 bp fragment including the full 16S, the intergenic sequence (ITS), and a portion of the 23S rRNA gene. The kit contains a patented mix of forward and reverse primers. The consensus sequence of the primers is 5′-AGRRTTYGATYHTDGYTYAG-3′ (forward) and 5′-AGTACYRHRARGGAANGR-3′ (reverse). Briefly, 10 uL 2X PCR Premix and 2 ng DNA were added to each well of the supplied Shoreline 96 well plate containing all primers and barcodes. PCR products were quantified (Qubit Broad Range kit, Invitrogen, Carlsbad, CA, USA) and quality control performed by an Agilent Fragment Analyzer (Agilent, Santa Clara, CA, USA). Individually barcoded amplicons were pooled and cleaned twice with 0.6 volumes of magnetic beads. The cleaned pool was checked on an Agilent Fragment Analyzer before library production.

The cleaned pool was converted to a barcoded PacBio library with the SMRTBell Express Template Prep kit 2.0 (Pacific Biosciences, Menlo Park, CA, USA). The library was sequenced on a SMRTBell 8M in the PacBio Sequel IIe using the CCS sequencing mode and a 15 h movie time. Demultiplexing of the PacBio library was performed using SMRT link V11 (Pacific Biosciences, Menlo Park, CA, USA). Demultiplexing of the Shoreline amplicons was performed using SBAnalyzer V3.0 (Shoreline Biome, Farmington, CT, USA).

16S and metagenomic data analyses were completed by the High-Performance Computing in Biology group at the University of Illinois. Shoreline amplicon data were quality-assessed using FASTQC [25] and then processed using a custom PacBio-adapted version of the TADA Nextflow-based workflow [26], which implements the DADA2 workflow [27] for dereplicating and denoising reads to generate single-nucleotide resolution Amplicon Sequence Variants (ASVs).

In brief: raw FASTQ data were demultiplexed using Shoreline SBAnalyzer v3.1-2 (https://intusbio.com), retaining the primer sequences. Sequences less than or equal to 1800 nt underwent primer sequence removal (FWD = “AGRGTTYGATYMTGGCTCAG”, REV = “RGYTACCTTGTTACGACTT”) and minimal quality trimming. Only sequences with a minimum of 1000 nt, a maximum expected errors (EE) score of 2, and no uncalled bases (‘N’) were retained. Error estimation included the additional parameters: “errorEstimationFunction = PacBioErrfun” and “BAND_SIZE = 32”. Default steps were used to denoise reads and dereplicate into ASVs, followed by taxonomic assignment using the DADA2 implementation of the RDP Classifier [28] and the Silva v138 database [29] custom formatted by DADA2 developers [30] for the optimization of long read data. Multiple sequence alignment of the resulting ASV sequences was performed by DECIPHER [31] followed by a midpoint-rooted Fasttree [32] phylogenetic analysis to produce a maximum likelihood tree used in subsequent data analysis steps.

Raw counts, taxonomic assignments, and the phylogenetic tree for the 27,975 ASVs were imported into R v. 4.0.3 [33] using the package phyloseq v. 1.34.0 [34]. Initially, minimal filtering was performed to remove mitochondrial and chloroplast ASVs and ASVs that were unassigned at the Phylum level, leaving 27,889 ASVs. Within each sample type, ASVs with zero counts and phyla with ASVs present in only one sample were removed. ASVs were tip-agglomerated at a phylogenetic distance <0.05, adding their counts together, followed by prevalence filtering at a threshold of 7.5% (present in two samples). The remaining ASVs were included in subsequent analyses.

### 2.5. Statistical Analysis

Statistical analysis was performed in R [33] version 4.3.1. Significance was set at *p* ≤ 0.05. Physical examination parameters, laboratory values, weight, and quantitative gastric fluid measurements (volume and pH) were evaluated for normality by the Shapiro–Wilk test and visual examination of q–q plots and histograms. Normally distributed data were summarized as mean ± SD and nonparametric data were summarized as median (Q1, Q3). Outliers were identified using the rstatix package version 0.7.2 [35] and removed. The effect of housing location and horse on numerical variables was determined by two-way ANOVA. The effect of housing location on categorical variables was determined by Chi Square test. Isolated microbial DNA concentrations were compared between sample types and housing locations by Kruskal–Wallis test with post-hoc Wilcoxon test with Bonferroni correction in the rstatix package version 0.7.2 [35].

Core ASVs in the gastric dataset were identified based on prevalence, and the relationship between prevalence and abundance, ignoring overall ASV abundance, based on the recommendations of Salonen et al. [36].

Alpha diversity analyses (Chao1, Shannon, and Simpson) were performed using the R packages phyloseq and vegan v. 2.5-7 [37]; Wilcoxon rank sum test was performed to assess differences between sample type and housing location. Beta diversity analyses were performed in R with phyloseq and vegan using relative proportion normalization to maintain community structure, and with both Bray–Curtis and Weighted Unifrac diversity metrics. Sample clustering was visualized using multidimensional scaling with Bray–Curtis [38] and Weighted Unifrac distances [39]. PERMANOVA [40], using the vegan function ‘adonis2′, was used to assess significance of differences in microbial community composition based on Bray–Curtis distance between sample type, horse, housing location, and interaction terms. Within each housing period and for both sample types, the effect of study week, horse, and horse*week interaction on Bray–Curtis distance was evaluated by two-way PERMANOVA. Where a significant difference was identified, pairwise PERMANOVA between factor levels was performed with multiple comparison adjustment by FDR.

Differential abundance analysis was performed using the DESeq2 package version 1.40.2 [41] to identify ASVs in gastric and fecal samples that were differentially abundant between housing conditions. The “poscounts” method was used for size factor estimation, which normalizes abundance by library size and per-taxon read depth to compensate for gross differences in composition between samples. The likelihood ratio test (LRT) was performed to identify ASVs with significant differential abundance among housing conditions and timepoints within housing conditions. The full model was Abundance = Horse + Housing + Timepoint. To test the effect of housing, the full model was compared to the model Abundance = Horse. To test the effect of timepoint within study period, the full model was compared to the model Abundance = Horse + Housing. Dispersions were fit using the “local” method, and significantly differentially expressed ASVs were considered at FDR < 0.05 using the “independent filtering” method of DESeq2.

## 3. Results

### 3.1. Horses and Monitoring

Horses ranged in age from 8 to 22 years old. Represented breeds were Standardbred (2), Quarter Horse (2), Thoroughbred (1), and American Warmblood (1). Five horses had ideal body condition scores (BCS 4–6/9) and one horse was over-conditioned (BCS 8/9). Horse signalments are included in Table S1. All horses completed the study without complications. Physical examinations were within normal limits for all horses at all time points, aside from the final sample collection (P4), at which time one horse (AN) was identified to have a superficial corneal ulcer, and a second horse (VA) was found to have pyometra. CBC parameters and fibrinogen remained within normal limits for all horses throughout the study.

Horse weight and physical exam parameters are summarized in Table 1. Weight varied significantly between horses (F = 759.3, *p* < 0.001), was higher during pasture than stable periods (F = 91.5, *p* < 0.001), and there was a significant Location*Horse interaction effect (F = 3.2, *p* =0.02). There was a statistically, but not clinically, significant increase in heart rate (F = 4.2, *p* = 0.05) and respiratory rate (F = 11.3, *p* = 0.002) during stable compared to pasture periods. The PCV differed significantly between individual horses (F = 2.7, *p* = 0.04), but not between housing locations. There was no significant effect of location or horse on rectal temperature or TS (Table S2).

### 3.2. Qualitative Description of Samples

Gastric fluid was successfully obtained from all horses at all time points using the double-tube technique; descriptive data are summarized in Table 2. There was no effect of housing location or horse on the fluid volume or pH (Table S2). There was a significant difference in gastric fluid color between study periods (*p* < 0.001), with samples collected from stabled horses significantly more likely to be yellow and less likely to be green. There was also a significant difference in gastric fluid quality (*p* = 0.02), with pasture samples more likely to be classified as “fibrous” and stable samples more likely “liquid”. There was no significant difference in subjective fecal color (*p* = 1) or moisture (*p* = 0.3) between study periods.

**Figure 2:**
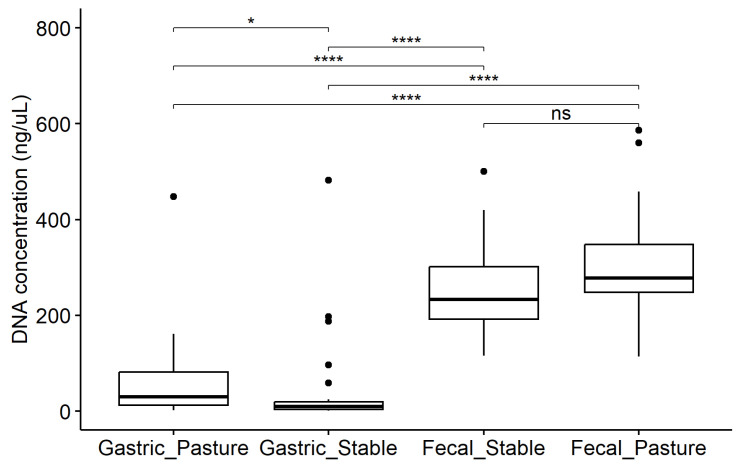
Box plots illustrating the measured concentration of isolated DNA; samples grouped by sample type and housing location. Kruskal–Wallis test showed a significant effect of group on DNA concentration (*p* < 0.001). Pairwise comparisons are indicated by brackets (ns, not significant; *, *p* < 0.05; ****, *p* < 0.0001).

The concentration of isolated DNA was 13.5 (5.7, 64) ng/uL in gastric samples, compared to 252.0 (213, 319) ng/uL in fecal samples. DNA concentrations (Figure 2) varied significantly between sample types and housing locations (*p* < 0.001), with significant differences for all pairwise comparisons other than the comparison between housing locations for fecal samples (*p* = 0.06).

### 3.3. Overall Microbiota Composition

Initially, minimal filtering was performed to remove mitochondrial and chloroplast ASVs, and ASVs that were unassigned at the phylum level leaving 27,889 ASVs. Within the gastric samples, removal of ASVs with zero counts left 4382 ASVs, and removal of phyla with ASVs present in only one sample further reduced the total to 4355 ASVs. Tip agglomeration and prevalence filtering resulted in 770 gastric “taxa” for statistical analysis (Figure S1a). Within fecal samples, removal of ASVs with zero counts left 24,894 ASVs, and removal of phyla with ASVs present in only one sample further reduced the total to 24,892 ASVs. Tip agglomeration and prevalence filtering left 5284 fecal “taxa” for statistical analysis (Figure S1b).

**Figure 3:**
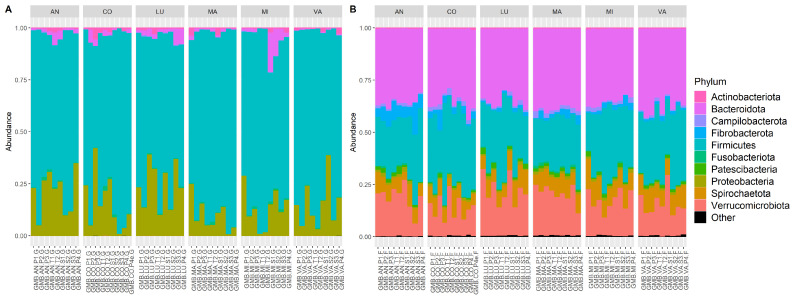
Relative abundance of the top 10 phyla in (**A**) gastric and (**B**) fecal samples by individual animals (AN, CO, LU, MA, MI, and VA), with time point along the x axis (P = pasture, T = transition, S = stabled), and relative abundance on the y axis.

**Figure 4:**
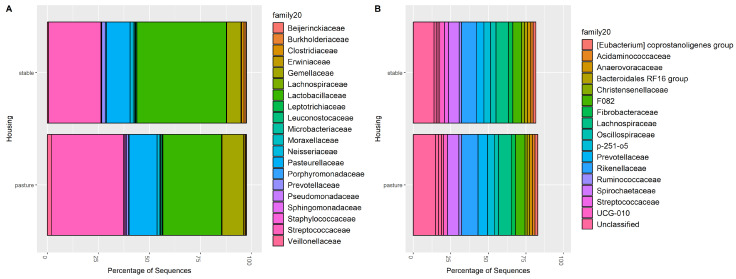
Percent abundance (number of taxa in each family/total sequences in each sample type) of the top 20 families in (**A**) gastric and (**B**) fecal samples.

The relative abundance of the top taxa fluctuated across individual horses and sample types (Figure 3). Dominant phyla in gastric samples were *Firmicutes* and *Proteobacteria*, while dominant phyla in fecal samples were *Bacteriodota*, *Firmicutes*, and *Verrucomicrobiota*. The identity of the top families was consistent between stable and pasture conditions for both gastric and fecal samples (Figure 4). In gastric samples, there was a relative increase in the proportion of *Lactobacillaceae* and decrease in *Streptococcaceae* when horses were stabled compared to samples collected during pasture housing.

### 3.4. Core Gastric Microbiome

**Figure 5:**
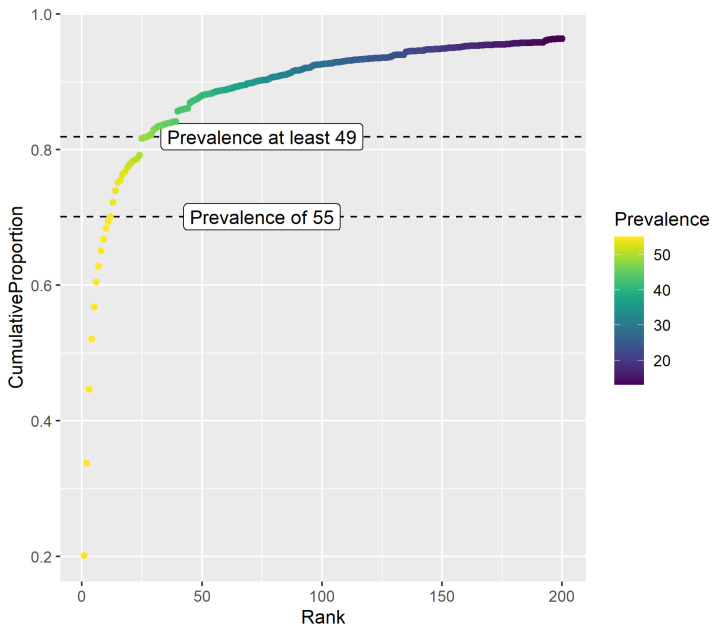
Cumulative proportion of reads for taxa ranked first by prevalence, then abundance. Dashed lines indicate suggested thresholds for core sets based on prevalence only (prevalence of 55), or the relationship between prevalence and abundance (prevalence of at least 49).

**Figure 6:**
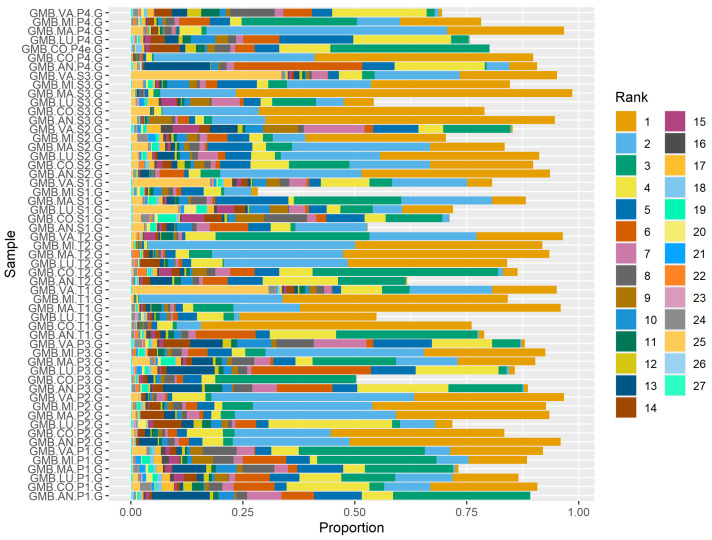
Within-sample abundance (proportion) of core taxa. Sample identity on the y axis is ordered by sample period first (P = pasture, T = transition, and S = stable), then individual horse (AN, CO, LU, MA, MI, and VA). The colors reflect the ranks of the taxa; description of taxa included in Table 3.

Twelve taxa were present in all 55 gastric samples, accounting for 73.9% of all sequencing reads. Based on the relationship between prevalence and abundance (Figure 5), taxa present in 49 samples could be considered for inclusion in a core microbiome, accounting for 83% of all sequencing reads (Table 3). Abundance of core taxa shows considerable subjective variation between samples (Figure 6). For example, grossly different abundances are apparent in the 1st and 2nd most abundant taxa, both *Lactobacillus* spp. (orange and light blue), as well as the 3rd and 4th top taxa, both *Streptococcus* spp. (green and yellow).

### 3.5. Diversity Metrics

#### 3.5.1. Alpha Diversity

Alpha diversity represents the richness and/or evenness of microbial species within a sample. Alpha diversity indices were calculated from agglomerated data before removal of low-prevalence taxa by filtration, as the presence of singletons/doubletons is considered in richness estimation. The selected alpha diversity metrics were the Chao1, Shannon, and Gini–Simpson indexes. The Chao-1 index reflects species abundance only and is underpinned by an assumption that the number of organisms within every taxa have a Poisson distribution, so it is especially useful for data sets skewed towards low-abundance taxa. The Shannon and Gini–Simpson indexes reflect both species richness and evenness. The Gini–Simpson diversity index gives more weight to dominant species and is less likely to be affected by a few rare species; when reported as Gini–Simpson index, a lower value means lower diversity. The Shannon index measures uncertainty of capturing similar species during random sampling, and as for the Gini–Simpson index, a lower value means lower diversity.

**Figure 7:**
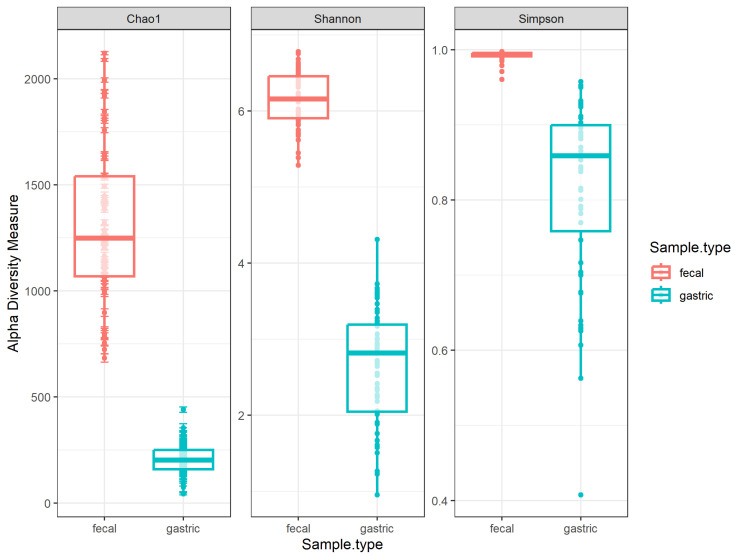
Boxplots with overlay scatter showing calculated alpha diversity metrics for gastric (blue) and fecal (red) samples. The Simpson index in this report is a Gini–Simpson index (1-D). Wilcoxon rank sum test was used to compare each metric between sample types (all *p* < 0.001).

Alpha diversity was first compared between housing locations within each sample type. There was no significant difference between pasture and stable housing for any metric (*p* > 0.2, Figure S2). When alpha diversity was compared between gastric and fecal samples, there was a significant difference between sample types for all three metrics (Figure 7). The Chao1 index was significantly lower in gastric fluid compared to fecal samples (*p* < 0.001), reflecting the overall lower abundance of microbial taxa in gastric fluid. The Shannon and Gini–Simpson indices were greater in fecal samples compared to gastric fluid (*p* < 0.001), suggesting that fecal microbiota were more diverse.

#### 3.5.2. Community Analysis

**Figure 8:**
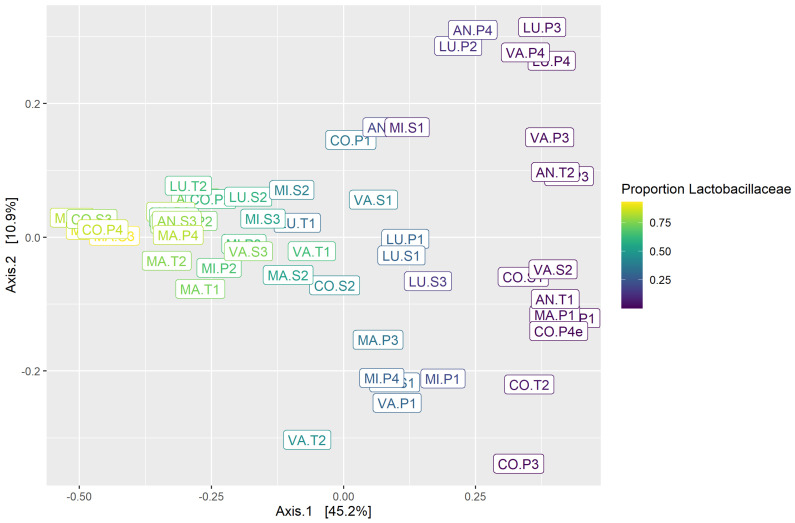
MDS plot of the gastric samples based on the Bray–Curtis distance, which is a measure of community structure. Color reflects the proportion of Lactobacillaceae, which was correlated with Axis 1.

**Figure 9:**
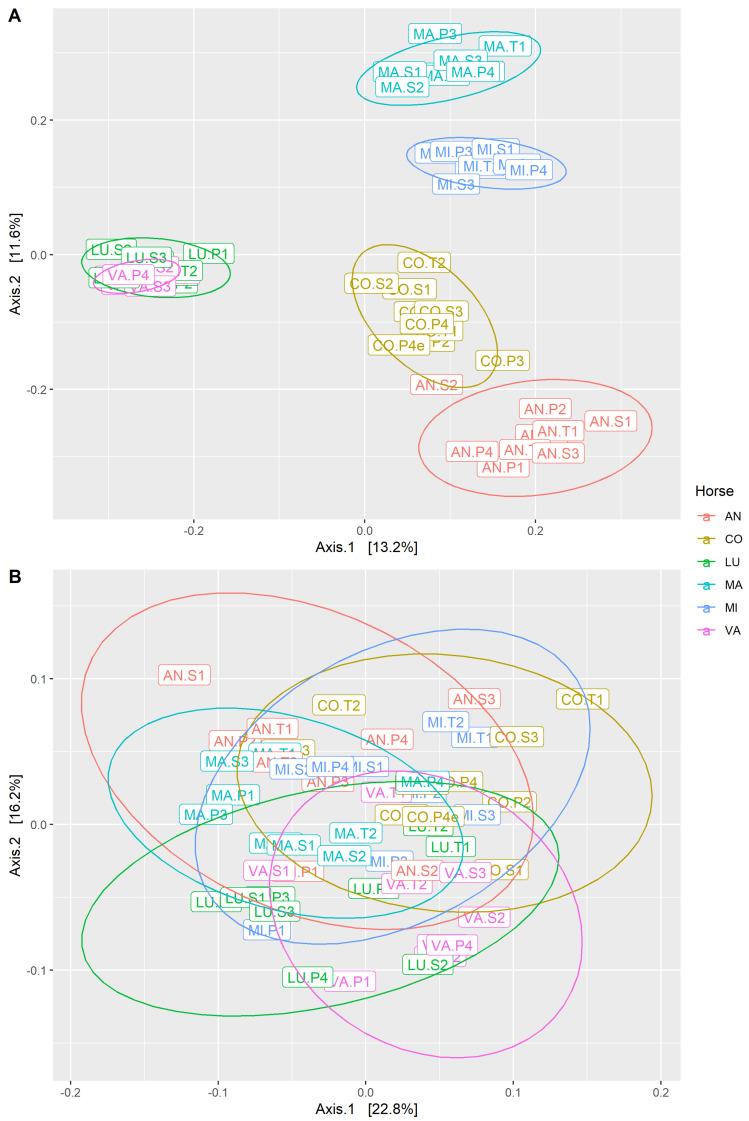
MDS plots of fecal samples based on the (**A**) Bray–Curtis distance and (**B**) Weighted UniFrac distance, which are both measures of community structure. Color reflects the individual horse identity, which demonstrated clear segregation between samples. Horse characteristics are described in Table S1; there were no apparent common traits between horses LU and VA to explain the significant overlap. Ellipses colored by horse identity represent a 95% confidence level based upon multivariate t-distribution.

**Figure 10:**
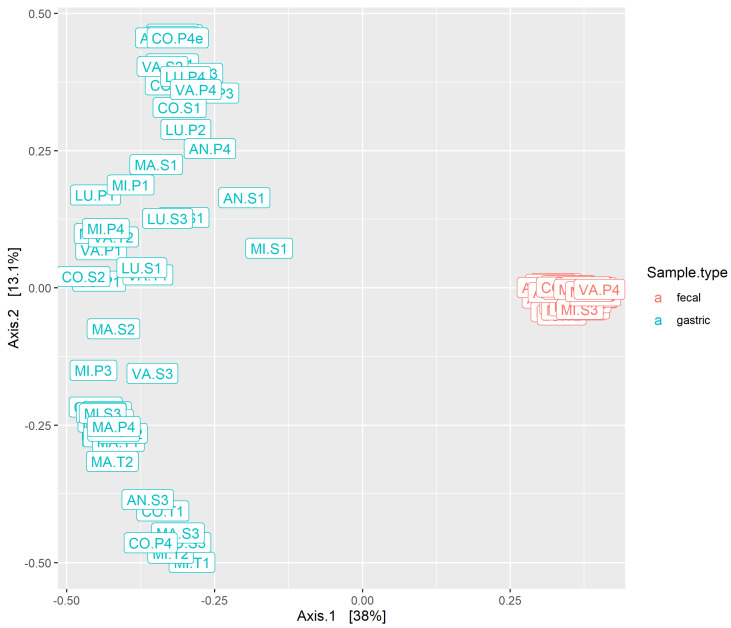
MDS plot of all samples based on the Bray–Curtis distance. Color reflects the sample type.

MDS plots based on Bray–Curtis distance were used to visualize differences in the community structure between samples. Within gastric samples, there was no apparent clustering by horse or by study period; however, the first axis was correlated with the abundance of Lactobacillaceae (Figure 8), which was subjectively observed to be variable between samples (Figure 6). Within fecal samples, there was clustering by horse but not by study period (Figure 9A). When gastric and fecal samples were analyzed together, there was strict clustering by sample type (Figure 10). The first axis was correlated with sample type, while the second axis varied among gastric samples, likely reflecting segregation by the proportion of Lactobacillaceae.

The MDS plots of the Weighted UniFrac distance, which accounts for phylogenetic relationships between taxa as well as the relative abundance, reflected the same findings as the plots of the Bray–Curtis distance, with one notable exception. Within fecal samples, the Weighted UniFrac distance did not display the same degree of clustering by horse as seen for the Bray–Curtis distance (Figure 9B). The MDS plots for both the Bray–Curtis and Weighted UniFrac distances, with multiple coloring schemes, can be viewed in Figure S3 (gastric) and Figure S4 (fecal).

PERMANOVA is a phylogenetic-distance-based method to test the association between microbial composition and covariates of interest. There was a significant effect of sample type on the Bray–Curtis distance (*p* = 0.001; Table 4). Within gastric microbiota, there was a significant effect of both housing location (*p* = 0.005) and horse (*p* = 0.009). A post-hoc pairwise PERMANOVA found no statistically significant distance between gastric microbiota of individual horses (all FDR ≥ 0.1; Table S3). Within fecal microbiota, there was a significant effect of housing location (*p* = 0.001), horse (*p* = 0.001), and location:horse interaction (*p* = 0.03). Post-hoc comparisons showed a significant difference between every pair of individual horses (all FDR ≤ 0.002; Table S4). Within each housing location and sample type, a two-way PERMANOVA found no significant effect of study week (fecal *p* > 0.09, gastric *p* > 0.9), while the significant effect of horse persisted (all *p* < 0.02; Table S5).

### 3.6. Differential Abundance

Interactive plots of differential abundance results can be visualized at https://doi.org/10.13012/B2IDB-7053728_V1; significant results will be highlighted here. Taxa not classified at the species level will be identified by the taxa id. Among the gastric microbiota, a total of 43 taxa were differentially abundant between housing locations (Table S6). *Streptococcus sp.41*, *Streptococcus sp.31*, and *Moraxella sp.3* had significantly greater abundance when horses were housed in stables, and returned to the lower, pre-stabling abundance when horses returned to pasture. However, other *Streptococcus sp. (20, 27, 37, 38, 39,* and *43)*, as well as *Pasteurellaceae sp.25* and *Veillonella sp.7*, had generally greater abundance in horses at pasture, decreased during stabling, and had returned to baseline at the final pasture sample collection. *Pasteurellaceae sp.24* abundance decreased during stabling and remained low in the final pasture sample. *Variovorax sp.1*, *Sphingomonas sp.6*, and *Leptotrichia sp.3* abundance increased markedly during the first two weeks of stabling (transition period) but returned to baseline for the remainder of the study. *Clostridium sensu stricto 1 sp.* was present in low abundance in few of the initial pasture samples, had markedly increased abundance during stabling, and was not detected in any of the final pasture samples.

Eleven taxa were differentially abundant in gastric samples among study weeks within housing locations (Table S6). *Actinobacillus equuli.4* had lower abundance in the initial weeks of stabling (T1, T2) and final pasture sample (P4) compared to other time points. *Curtobacterium sp.* increased during the stabling period, with peaks at T1 and S1, then sharply decreased at S2 and S3. *Alysiella sp.1* had peaks in abundance at the end of pasturing (P3) and the middle of the stabling (S1) period, before progressively decreasing for the remainder of the study. *Prevotella sp.37* had fluctuating abundance, which was greater at time points P2, T2, and P4 compared to other sampling times. *Veillonella sp.7* showed a similar pattern, with greater abundance at P2 and P4. Some taxa had significantly greater abundance in one week compared to all other time points: *Pasteurellaceae sp.33* in the second week of pasturing (P2) and *Pantoea sp.2*, *Pantoea sp.1*, and *Methylobacterium-Methylorubrum adhaesivum.2* in the first week of stabling (T1).

Among the fecal microbiota, a total of 38 taxa were differentially abundant between housing locations (Table S7). The abundance of *Lactobacillus hyakatensis* was lower during the stabling period than initial pasture period and remained decreased at the final pasture sample collection. *Prevotellaceae UCG-001 sp.2* and *Lachnospiraceae AC2044 group sp.49* abundance decreased markedly during stabling but returned to pre-stabling abundance in the final pasture sample. *Treponema sp. 106*, *Treponema sp.79*, *Treponema saccharophilum.4*, and *Bacteriodales UCG-001 sp.10* decreased during the first two weeks of stabling (T1 and T2) and then quickly increased to or above the initial abundance for the remainder of the study. On the other hand, *Streptococcus sp.13* abundance was high throughout the stabling period and was not detected in initial or final pasture samples. *F082.11* and *Bacteroidales UCG-001 sp.7* were not consistently found in all individuals, but when present had greater abundance during pasture periods than stabling, where it was identified in only five samples over the 5 weeks. *Weissella sp.* was only present in two samples during pasturing (P2 and P4) but was found in the majority of samples during the stable period. *Colicodextribacter sp.4* was absent from all but two of the initial pasture samples and found in very high abundance in the first week of stabling (T1), followed by lower abundance for the remainder of the study.

Five taxa were differentially abundant in fecal samples between weeks within housing locations (Table S7). *Treponema sp.79* increased during the initial pasture period, decreased during the first weeks of stabling and then increased again to peak at week S2 before decreasing once more. *Lactobacillus equigenerosi* peaked at the second week of the study (P2), and *Lactobacillus hyakatensis* peaked at the first week of stabling (T1), before each gradually decreased over subsequent weeks. *Streptococcus sp.13* increased over the first three pasture weeks, increased markedly at the first stable week (T1), and remained at high abundance until the final pasture week. *Rikenellaceae RC9 gut group sp.144* increased markedly in the 2nd week and then had variable abundance for the remainder of the study.

## 4. Discussion

Overall, the microbial community of gastric fluid from healthy horses contained less microbial DNA and fewer taxa compared to paired fecal samples. Species richness and diversity differed significantly between sample types, with greater abundance and a predominance of rare species in fecal microbiota, but not between housing locations. There was a significant difference in community composition (Bray–Curtis distance) between housing locations for both gastric and fecal microbiota, but no significant week-to-week variation within housing conditions, suggesting that stable gastric and fecal microbial populations are maintained under each management condition.

Fecal samples are often used to study the microbiome, given the ease of collection and availability, but have a distinctly different microbiome from luminal samples collected at the level of the foregut [20] and cecum [23]. As horses are hindgut fermenters with a long transit time, large volume of digesta, and relatively large numbers of bacteria present within the hindgut, effects seen in the stomach may be masked by the time ingesta reaches the level of the hindgut and therefore not reflected in the fecal microbiome [42].

To our knowledge, this is the first report of microbial profiling analysis from native gastric fluid collected from awake, non-fasted horses. Previous studies of the equine gastric microbiome have used samples obtained by gastroscopy or at post-mortem examination [20,23,43,44,45,46]. It is reasonable to expect that fasting (as required for gastroscopic examination) or death would affect the microbial population within the stomach [43], impacting characterizations of normal flora. Native gastric fluid can be easily obtained from standing horses without fasting or other intervention, making it an attractive option for investigation of the gastric microbiome in health and disease [47]. Previous equine studies have identified differences in microbial community composition between gastric mucosa and gastric fluid [20,44]. When gastric fluid was collected by tap water lavage, there was extremely variable microbial communities with no clustering by individual or treatment; fluctuation over time masked any effect of the intervention being studied [42]. Gastric fluid was readily obtained from all horses in this study using the described double-tube technique, without fasting or any other intervention prior to sample collection. While the volume and consistency of fluid obtained did vary between horses and over time, there was no effect of time or housing condition on the sample pH.

On the contrary, findings presented here support the presence of a stable gastric fluid microbial community in healthy horses. The microbial community of gastric fluid collected from fasted horses [44] found the same top three phyla (Proteobacteria, Firmicutes, and Bacteroidetes), although in fasted horses Proteobacteria predominated while Firmicutes predominated in the non-fasted horses in our study. Withholding feed has been demonstrated to affect microbiota diversity and composition in feces of healthy horses [48].

This study focused on luminal microbial communities, rather than mucosal communities. In other areas of the gastrointestinal tract, comparison of luminal to mucosal microbial communities have found increased richness in mucosal samples [49] and differences in community structure [21]. These findings contrast with a previous study, which found similar mucosal and luminal populations within the stomach and small intestine when resolved to the OTU level [20]. However, that study did report a greater difference between luminal microbial communities of the small and large intestine, suggesting that comparison of luminal samples is worthwhile.

Within the gastric samples, 74–83% of sequencing reads were attributed to core taxa depending on the threshold selected for consideration. This proportion is much greater than core populations reported from other regions of the equine intestine, where core populations in the ileum account for 32% of all sequences and 5–15% of sequences in the large intestine [11]. The two most abundant members of the core community belonged to Lactobacillaceae (combined 34% of reads), similar to a previous report of the ileal core community [11]. Other families represented were *Streptococcaceae*, *Pasteurellaceae*, and *Gemellaceae*. In contrast, *Lachnospiraceae* and *Prevotellaceae* are consistently reported as dominant taxa within hind gut core microbiome [13].

Identifying core microbiota has been a goal of microbiome research since the beginning of the human microbiome project [50]. However, there is little consensus on quantification or even definition of “core microbiome”, and the metrics used may be susceptible to sampling or other biases, complicating interpretation. In this study, we have identified “core microbiota”, defined as the microbial taxa in common within a particular environment. The “core microbiome” would more accurately incorporate community structure and function as well as abiotic conditions [51]. Technical factors (e.g., sequencing depth, primer set, and sequencing length), study design choices (spatial and temporal scale of sampling collection), and methods of taxa inclusion (occurrence- vs abundance-based criteria) influence core microbiota results and limit the ability to compare between studies [52,53]. While investigations of the core microbiome have largely focused on taxonomic identity of member microbiota, the core may be better defined from a functional perspective rather than identity [39]. The concept of a functional core microbiome suggests that individuals may have different taxa or ASVs fulfilling the same functional role within the microbial ecosystem; incorporation of metabolomics and metagenomics data into future analyses may provide a more functionally relevant definition of the core microbiome [54].

While the impact of extreme diet change on intestinal microbiota has been well documented, effects of seemingly innocuous husbandry changes (such as from pasture- to hay-based forage diets) are less well studied. It is not surprising that the sensitive microbial community structure within the equine gastrointestinal tract would be susceptible to such an apparently minor change. This is highly relevant to the design of controlled interventional studies, as horses in research herds are commonly maintained on pasture turnout between protocols but are stabled during experimental trials for logistical purposes. As there appears to be a transient effect on the microbiome during the transition from pasture to stable, it is critical for researchers to allow time for the microbiome to equilibrate before initiating experimental protocols.

There was a relative increase in proportion of *Lactobacillaceae* and decrease in *Streptococcaceae* in gastric fluid when horses were stabled compared to pasture housing. *Lactobacillaceae* are lactic-acid fermenting bacteria, commonly identified in equine intestinal contents. Increased relative abundance of fecal *Lactobacillaceae* has been reported in conjunction with colic [55], colitis [49,56], and maturation of foals [57]. Overall, increases in fecal *Lactobacillus* and *Streptococcus* have been considered negative changes in microbial flora [58]; the relationship between gastric abundance of these families and horse health is less clear.

*Streptococcaceae* are more abundant in feces when horses are fed a high starch diet [59] and less abundant when fed the most mature hay [60]; the relative decrease when horses moved into stables may reflect the removal of fresh grass from the diet. *Streptococcus sp.* are also associated with carbohydrate overload, both in cecal flora in vitro [61] and in feces from horses with oligofructose-induced laminitis [62], suggesting a possible connection to pasture-associated laminitis. However, a study of gastric mucosal microbiota found that streptococcus predominated in samples from horses that were stabled, fed hay, and sampled post-mortem, but not in horses pastured with hay and grass and sampled by gastroscopy [43]. *Streptococcaceae* are increased in gastric fluid humans after omeprazole administration [63], illustrating a possible relationship with gastric pH. In differential abundance analysis there were unclassified *Streptococcus* species with greater abundance at pasture, and others with greater abundance during stabling, within both gastric and fecal samples. This finding is likely attributable to the heterogenous nature of the genus and is an example where functional evaluation may have greater utility than taxonomic classification.

*Lactobacillus equigenerosi* and *Lactobacillus hyakatensis* were differentially abundant in the fecal samples, with a peak when horses were first moved into the stable, followed by lower abundance during stabling. As *Lactobacillus* overgrowth has been associated with colic [55], this transient increase suggests a possible association with colic related to changes in housing. *L. equigenerosi* was also enriched in biopsy samples from equine glandular gastric disease lesions [46] and has been shown to invade intestinal epithelial cells [64], further supporting a possible negative effect on gut health during the transition from pasture to stable housing. However, none of the horses in this study showed signs of colic, so further study would be necessary to substantiate this connection.

Unclassified species belonging to *Pasteurellaceae* and *Veillonella* had greater abundance in gastric fluid when horses were at pasture compared to stabling. *Pasteurellaceae* are primarily commensal species that colonize mucosal surfaces [65] and have been reported as one of the major families of the quine stomach [20,43,66,67] and small intestine [11]. *Veillonella* is one of the primary lactate-utilizing microbes in the equine gastrointestinal tract [68], and its abundance has been related to dietary starch content [5,69]. A stable *Veillonella* population appears to be reflective of intestinal health, as abundance is decreased following oxytetracycline administration [70] and grossly increased in horses with equine grass sickness [71].

Several taxa were differentially abundant between study periods with significantly greater abundance in gastric fluid during the initial weeks of stabling only (“transition period”). The majority of these are taxa not typically reported as intestinal microbiota. *Variovorax* are found primarily in soil and fresh water [72,73], and have been reported in feces of tree shrews [74] and humans with concurrent *Clonorchis siensis* parasitic infection [75]. *Curtobacterium* are typically plant pathogens or symbionts involved in carbohydrate degradation [76,77]. Likewise, *Methylobacterium-Methylorubrum adhaesivum* is found in plant endophytes [78,79] and water [80]. *Pantoea sp.* are also isolated from soil and plants [81] and appear to be opportunistic pathogens in horses, as reported in ulcerative keratitis [82] and fibronecrotic placentitis [83]. With the exception of *M. adhaesivum*, which has been reported as a contaminant of DNA extraction kit reagents [84], it is likely that these taxa were transient members of the gastric microbiota acquired from the environment.

A second group of taxa with the pattern of increased abundance during the transition to stabling are more typically associated with the oral cavity or upper respiratory tract. *Leptotrichia sp.* have been previously reported in equine subgingival plaque [85] as well as paired gingival swabs and post-exodontia blood samples [86]. In Mongolian horses, *Leptotrichia* were more abundant in the stomach compared to other parts of the gastrointestinal tract [67]. *Alysiella sp.* are also part of a family found primarily in the oral cavity [87,88], including that of donkey foals [89]. *Sphingomonas* is a diverse genus which includes environmental bacteria, opportunistic pathogens, and commensal microbes [90]. *Sphinogmonas* species have previously been reported as a top taxa in weanling gastric contents [91] and are abundant taxa in the equine ocular and respiratory microbiomes [92,93].

Two taxa had significantly greater abundance during stabling compared to pasture. *Moraxella* is highly abundant in equine subgingival plaque [85], and increased abundance in duodenal contents was reported when horses were fed a high starch diet [94]. However, *Moraxella* is also very common in the upper respiratory tract of cattle [95], and it is worth noting that the facility used to house the horses during stabling in this study also houses cattle. *Clostridum sensu stricto*, the “true” clostridium genus containing important pathogens [96], also markedly increased during stabling. Increased abundance of *Clostridum sensu stricto* in cecal contents has been reported in horses with diarrhea [97], and it is found in greater abundance in the stomach than large intestine [67].

*Prevotellaceae* and *Lachnospiraceae*, both putatively beneficial members of the fecal microbiota, had significantly reduced abundance in fecal samples during the stabling period. *Prevotellaceae UCG-001* was found in higher abundance in yak when grazing pasture compared to stabling [98]. Evidence for possible beneficial effects include the negative correlation with oxidative stress in sows [99] and positive correlation with feed efficiency in cattle [100]. *Lachnospiraceae AC2044 group* are butyrate producers, which is protective for enterocytes [101]. The presence of *Lachnospiraceae* in the fecal microbiota has been associated with healthy horses in multiple studies [49,56,102,103], while decreased abundance of both *Prevotellaceae* and *Lachnospiraceae* has been associated with colic [104].

Unclassifed taxa from families containing other putatively beneficial fecal microbiota, *Bacteriodota F082*, *Bacteriodales UCG-001*, and *Baterioidales BS11 gut group*, were not found in all individuals, but when present had greater abundance during pasture periods. These taxa have been shown to positively correlate with short-chain and branched-chain fatty acids, both thought to be beneficial for colonic health [105]. Bacteroidetes abundance was greater in obese compared to healthy body weight ponies [16]. Horses in this study had a range of body conditions, which may explain the presence of these taxa in only some individuals, although additional data are needed to substantiate this connection. *Rikenellaceae RC9 gut group*, another genus involved in carbohydrate degradation, was most abundant during the pasture period. Consistent with a previous study, taxa of this genus were variable between horses [106]. In bovids, *Rikenellaceae RC9 gut group sp.* has an important role in the digestion of crude fiber [98], and is more abundant when fed a lower starch diet [107], especially mature pasture [108].

On the contrary, unclassified *Weissella sp.* was found in the majority of fecal samples from the stable period but only two individual pasture samples. This taxa produces lactic, acetic, and short-chain fatty acids with multiple suggested beneficial effects [109]. Possible benefits include degradation of prebiotic oligosaccharides [110] and production of antimicrobial exopolysaccharides [111,112], thereby favoring growth of other probiotic species such as *Lactobacillus* [113]. A previous study found that supplementation of hay rations with sugar beet pulp resulted in increased abundance of *Weissella*, with the hypothesized effect of mitigating functional changes associated with removal from pasture [114]. However, removal from pasture without a change in hay rations in our study also resulted in increased *Weissella* abundance.

Another group of fecal microbiota including *Treponema saccharophilum*, unclassified *Treponema sp.*, and unclassified *Bacteroidales UCG-001* showed a pattern of decreased abundance in the early stabling period (T1-T2) followed by a rapid increase. Both *Treponema* and *Bacteroidales UCG-001* have been shown to fluctuate significantly when horses are fed reduced lignin alfalfa [115], which is a modification that improves digestibility. *Treponema* has also been associated with fecal particle size [115] and was enriched in healthy horses compared to horses with colitis [56]. Therefore, the decreased abundance of this group during the transition to stabling may contribute to the risk of digestive upset with changes in housing. However, no gastrointestinal distress was noted in this study to substantiate such a connection, and additional investigation is needed.

The primary limitations of this study were the small number of horses of a single sex and the potential effect of season. A repeated measures design was used to increase study power. Still, the small sample size may have decreased the statistical power to capture significant week-to-week differences. The cohort was limited to a single sex to reduce factors contributing to individual variability, as sex and reproductive status have been shown to impact microbial community composition [116]. The pasture grass and hay species were not identified or recorded, and it is possible that microbial populations would have responded differently to different grass species. Season and ambient weather conditions have been associated with changes in fecal microbiota composition [117]. Sample collection was completed from May to July in the midwestern United States, during which time the weather is consistently estival, so it is unlikely that seasonal changes confounded the results of this study. However, the effect of pasture/housing during a different season may differ.

## 5. Conclusions

The findings reported here support the presence of a stable microbial community within the gastric fluid of healthy horses, which is distinct from the fecal microbial community. The double-tube technique of collecting native gastric fluid may be used for future study of gastric fluid microbiota in diseases or therapeutic interventions affecting the foregut.

While the microbial community structure differed between pasture and stable housing, there was no significant week-to-week variation within each study period, suggesting that housing-associated changes stabilize within one week. These housing-related changes should be considered in the design of future gastric microbiota studies.

## Figures and Tables

**Table 1:** Physical exam and stall-side blood test values during pasture and stable periods of the study. Results reported as mean ± SD. Values in bold text were significantly different between housing locations.

Parameter	Pasture	Stable
Weight (kg)	**555.5 ± 67.1**	**541.4 ± 71.1**
Temperature (°C)	36.9 ± 0.6	37.2 ± 0.4
Heart Rate (beats/min)	**34.9 ± 5.7**	**37.4 ± 3.6**
Respiratory Rate (breaths/min)	**15.2 ± 1.9**	**12.9 ± 2.9**
Packed Cell Volume (%)	35.5 ± 4.3	34.4 ± 3.9
Total Solids (g/dL)	6.7 ± 0.9	6.8 ± 0.22

**Table 2:** Parameters for descriptive analysis of gastric fluid and fecal samples during pasture and stable periods of study. Quantitative variables are summarized as mean ± SD and qualitative variables are summarized by number of observations at each level.

		Pasture	Stable
**Gastric Fluid**			
Volume (mL)		12.0 ± 3.5	19.9 ± 21.1
pH		5.5 ± 1.5	5.9 ± 1.5
Color ^1^	Green	12	4
	Yellow	0	18
	Brown	12	8
Quality	Fibrous	13	7
	Liquid	9	23
**Feces ^2^**			
Color	Brown	12	17
	Green	8	13
Moisture	Normal	16	18
	Dry	2	4
	Soft	2	8

^1^ Color data was missing for *n* = 6 gastric fluid samples collected during pasture period. ^2^ Qualitative data was missing for *n* = 10 fecal samples collected during pasture period.

**Table 3:** Taxa for consideration in core gastric microbiome as ranked by prevalence. Taxa above the dashed line present in all 55 samples; based on relationship between prevalence and abundance, taxa present in ≥49 samples were considered for core microbiome inclusion. NA indicates taxonomic classification was not possible at the species and/or genus level.

Taxon MD5sum	Rank	Prevalence	Total Abundance	Cumulative Proportion	Family	Genus	Species
100050d9d6cc8552560217db2ff507a4	1	55	177,963	0.2012091	Lactobacillaceae	*Lactobacillus*	*hayakitensis*
b84694a3496a1b858e4e56abf0364056	2	55	120,563	0.3375204	Lactobacillaceae	*Lactobacillus*	*equigenerosi*
246167e02765e37c8611b7f941b300e8	3	55	96,001	0.4460614	Streptococcaceae	*Streptococcus*	NA
abf79ace8c371a4f8b5e939350245885	4	55	65,731	0.5203784	Streptococcaceae	*Streptococcus*	NA
e47660136989d61a8959fee1f374751d	5	55	42,181	0.5680692	Gemellaceae	*Gemella*	NA
83541fbb51ab03edf9cbf9fb71683ffc	6	55	32,156	0.6044255	Pasteurellaceae	NA	NA
2f912880e904a3e3c4f3a3e4e895f979	7	55	21,111	0.6282941	Pasteurellaceae	NA	NA
b377f42e15f0377ab7efca1840dabfaf	8	55	19,645	0.6505052	Streptococcaceae	*Streptococcus*	NA
7731f062d1f6ac374cc8638b74c9a2c4	9	55	15,230	0.6677246	Pasteurellaceae	*Actinobacillus*	NA
f80d643795a2c7227e8cbca85cb01933	10	55	13,969	0.6835182	Gemellaceae	*Gemella*	NA
3aa6b9c29ea3976f9d88e5bd7816a395	11	55	9142	0.6938544	Streptococcaceae	*Streptococcus*	NA
3bcf6d6537459f6a28b26c69f8353fc2	12	55	6208	0.7008733	Streptococcaceae	*Streptococcus*	NA
6b12ae81f8f05ad860d5ba8251f4cd5b	13	54	18,957	0.7223065	Gemellaceae	*Gemella*	NA
340cf14efd2146d16b31ad68e97e1436	14	54	15,199	0.7394909	Streptococcaceae	*Streptococcus*	NA
4a37050c8b0dbc74c2fed56852cae010	15	54	10,389	0.7512369	Streptococcaceae	*Streptococcus*	NA
f7119b783293c7f8c7168064942a3ac7	16	54	3480	0.7551715	Pasteurellaceae	*Actinobacillus*	NA
c09e7ed1453ae32158f71be7ac5d1573	17	53	7382	0.7635177	Pasteurellaceae	*Actinobacillus*	NA
60a3df9a52e5815152ac24d601051ac0	18	53	3420	0.7673845	Streptococcaceae	*Streptococcus*	NA
9a7de478261413c59925d107ce82609f	19	52	6059	0.7742349	Neisseriaceae	*Alysiella*	NA
bdfb5770bef6e0414b89b16ff05bf56e	20	52	4026	0.7787868	Streptococcaceae	*Streptococcus*	*dentapri*
6a8ade15778d334c551849fc7d35ae4a	21	52	3259	0.7824715	Pasteurellaceae	*Actinobacillus*	NA
371fc770a6922f3f42de298930c8a4ad	22	51	2175	0.7849306	Leptotrichiaceae	*Leptotrichia*	NA
7228b9f0936a28ed5296ce2b08ae07f7	23	51	1215	0.7863043	Neisseriaceae	NA	NA
970b1261d3e7296fa04243ae0e07678f	24	50	4512	0.7914057	Pasteurellaceae	*Actinobacillus*	NA
401877543d15fdaeeea1aaec1bb86896	25	49	22,104	0.8163970	Lactobacillaceae	*Lactobacillus*	*equi*
c40bb8b34b97e17fec2005867c55eb12	26	49	1134	0.8176791	Gemellaceae	*Gemella*	NA
3fe20cb6437be80a6ec4ee015d10c635	27	49	1087	0.8189081	Streptococcaceae	*Streptococcus*	*dentasini*

**Table 4:** Results of the PERMANOVAs. PERMANOVA is a phylogenetic-distance-based method to test the association between covariates of interest using the Bray–Curtis distance.

PERMANOVA Model	Model Terms	Sum Squares	Df	*p* Value
Bray distance (all samples) ~ Sample type	Sample type	14.6	1	0.001 *
	Residuals	24.5	108	NA
Bray distance(gastric) ~ Location * Horse	Location	0.628	1	0.005 *
	Horse	1.72	5	0.009 *
	Location:Horse	0.715	5	0.7
	Residuals	7.32	54	NA
Bray distance(fecal) ~ Location * Horse	Location	0.466	1	0.001 *
	Horse	5.88	5	0.001 *
	Location:Horse	1.02	5	0.025 *
	Residuals	6.83	43	NA

Df = model term degrees of freedom, Location = housing location (pasture or stable), * indicates *p* < 0.05.

## Data Availability

The original data presented in the study are openly available in the Illinois Data Bank, https://doi.org/10.13012/B2IDB-7053728_V1.

## Supplementary Materials

The following supporting information can be downloaded at: https://www.mdpi.com/article/10.3390/ani14202979/s1, Figure S1: Prevalence vs abundance of represented families in (a) gastric and (b) fecal samples. Horizontal dashed lines indicate the threshold of 2 or more samples for prevalence filtering; Figure S2: Boxplots with overlay scatter showing calculated alpha diversity metrics for (a) gastric and (b) fecal samples. Wilcoxon rank sum test was used to compare each metric between housing location (pasture, red; stable, blue); Figure S3: MDS of gastric samples using (a–c) Bray Curtis distance and (d–f) Weighted UniFrac distance. Points colored by (a,b) study period, (c,d) horse, (e,f) proportion of Lactobacillaceae. Ellipses [colored by (a,b) study period and (c,d) horse,] represent a 95% confidence level based upon multivariate t-distribution; Figure S4: MDS of fecal samples using (a,b) Bray Curtis distance and (c,d) Weighted UniFrac distance. Points and ellipses colored by (a,b) study period, and (c,d) horse. Ellipses represent a 95% confidence level based upon multivariate t-distribution; Table S1: Signalment and body condition score1 (BCS) of horses in the study; Table S2: Results of ANOVA on physical examination and qualitative gastric fluid parameters. * denotes statistical significance; Table S3: Results of post hoc pairwise PERMANOVA for Bray Curtis distance between gastric microbiota of individual horses (AN, CO, LU, MA, MI, VA); Table S4: Results of post hoc pairwise PERMANOVA for Bray Curtis distance between fecal microbiota of individual horses (AN, CO, LU, MA, MI, VA); Table S5: Results of two-way PERMANOVA, a phylogenetic distance-based method to test association between study week, horse, and week:horse interaction on Bray Curtis distance within each sample type and housing location; Table S6: Results of differential abundance analysis for gastric fluid microbiota between housing locations (location) and study weeks (timepoint). Base mean = average normalized count values across all samples; Table S7: Results of differential abundance analysis for fecal microbiota between housing locations (location) and study weeks (timepoint). Base mean = average normalized count values across all samples.
