# Supplementary material for: Stability of Gastric Fluid and Fecal Microbial Populations in Healthy Horses under Pasture and Stable Conditions
Source: Animals (Basel). 2024 Oct 16;14(20):2979. doi: 10.3390/ani14202979 (PMC11503871; doi:10.3390/ani14202979)
Supplement: Supplementary file 1 [file animals-14-02979-s001.zip › animals-3241289-supplementary.pdf]

Table S1. Signalment and body condition score[1] (BCS) of horses in the study.

Horse ID	Breed	Age	BCS
AN	American Warmblood	20	5/9
CO	Standardbred	19	4/9
LU	Quarter Horse	8	8/9
MA	Standardbred	22	6/9
MI	Thoroughbred	16	5/9
VA	Quarter Horse	19	4/9

[1] BCS based on scale described by Henneke et al. {henneke 1983}; 4-6 is considered ideal.

Table S2. Results of ANOVA on physical examination and qualitative gastric fluid parameters. * denotes statistical significance.

	df	F	p-value
**Weight (kg)**			
Location	1	91.5	< 0.001 *
Horse	5	759.3	< 0.001 *
Location*Horse	5	3.2	0.02 *
**Temperature (°C)**			
Location	1	3.1	0.09
Horse	5	1.5	0.2
Location*Horse	5	1.9	0.1
**Heart rate (beats/min)**			
Location	1	4.2	0.05 *
Horse	5	1.0	0.4
Location*Horse	5	0.9	0.5
**Respiratory rate (breaths/min)**			
Location	1	11.3	0.002 *
Horse	5	0.3	0.9
Location*Horse	5	0.5	0.8
**Packed Cell Volume (%)**			
Location	1	1.1	0.3
Horse	5	2.7	0.04 *
Location*Horse	5	1.1	0.4
**Total solids (g/dL)**			
Location	1	0.9	0.3
Horse	5	0.3	0.9
Location*Horse	5	0.08	> 0.9
**Gastric fluid volume (mL)**			
Location	1	3.3	0.08
Horse	5	1.1	0.4
Location*Horse	5	1.1	0.4
**Gastric fluid pH**			
Location	1	0.4	0.5
Horse	5	0.3	0.9
Location*Horse	5	0.4	0.8

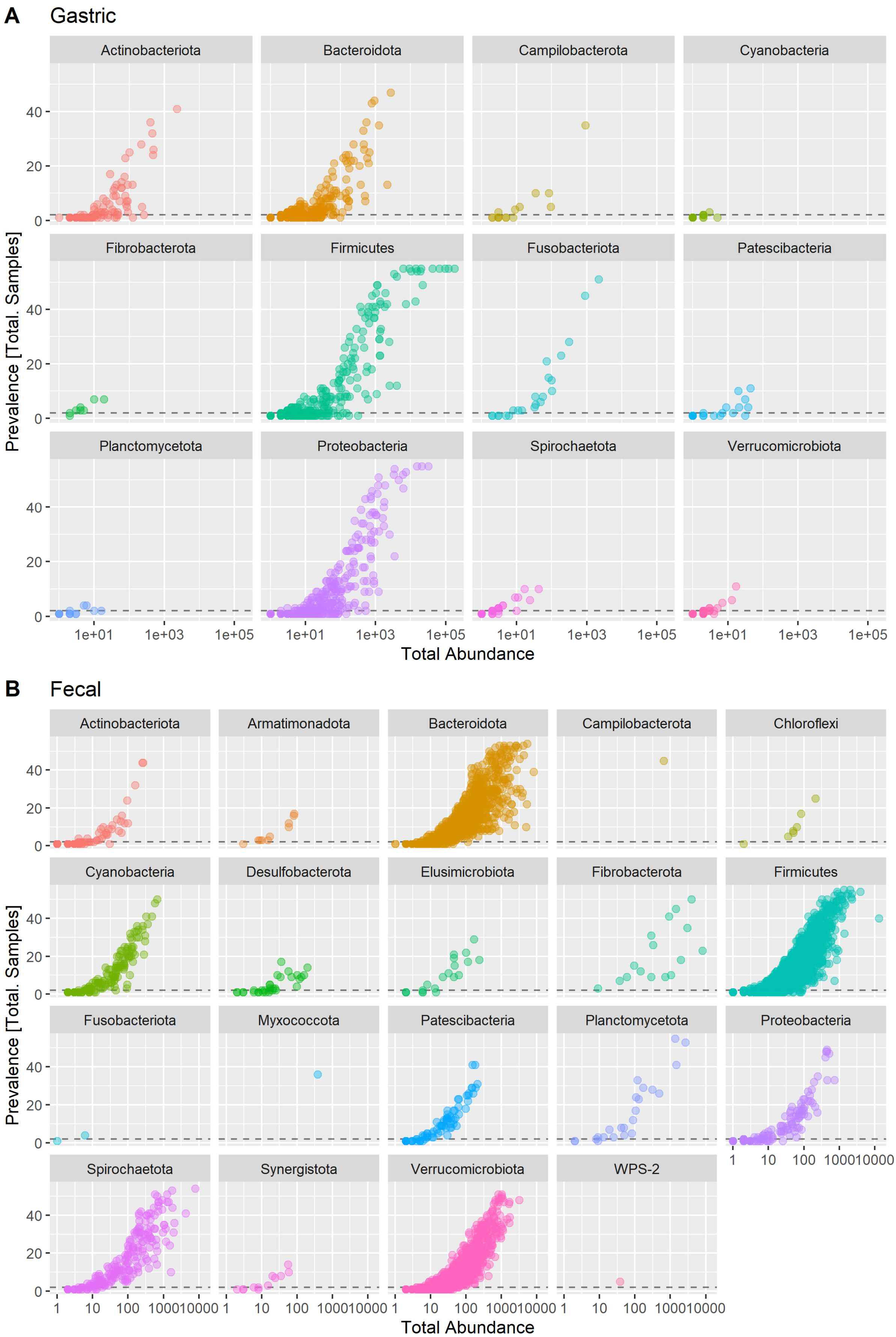

Figure S1. Prevalence vs abundance of represented families in (a) gastric and (b) fecal samples. Horizontal dashed lines indicate the threshold of 2 or more samples for prevalence filtering.

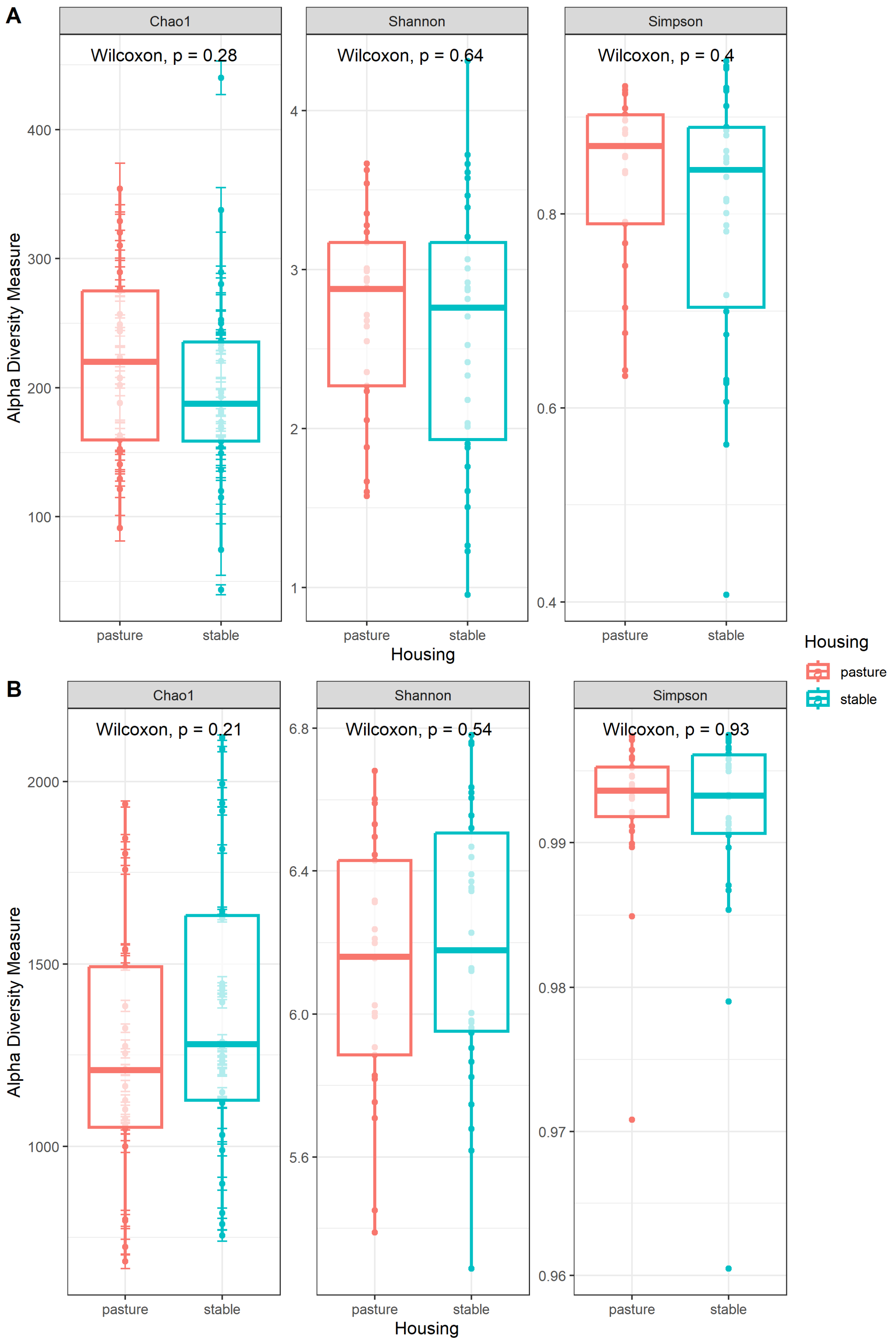

Figure S2. Boxplots with overlay scatter showing calculated alpha diversity metrics for (a) gastric and (b) fecal samples. Wilcoxon rank sum test was used to compare each metric between housing location (pasture, red; stable, blue).

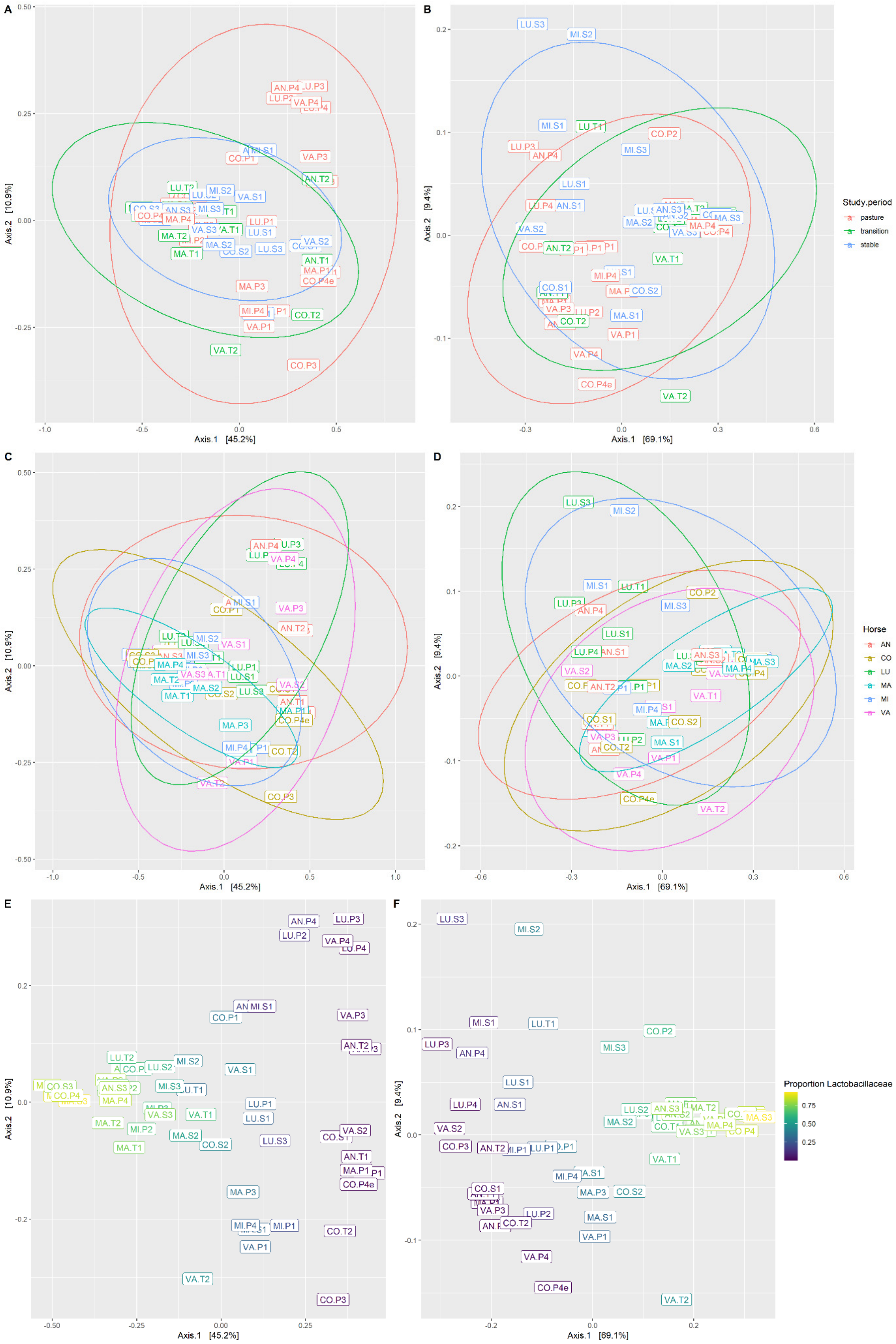

Figure S3. MDS of gastric samples using (a-c) Bray Curtis distance and (d-f) Weighted UniFrac distance. Points colored by (a, b) study period, (c, d) horse, (e, f) proportion of Lactobacillaceae. Ellipses [colored by (a, b) study period and (c, d) horse,] represent a 95% confidence level based upon multivariate t-distribution.

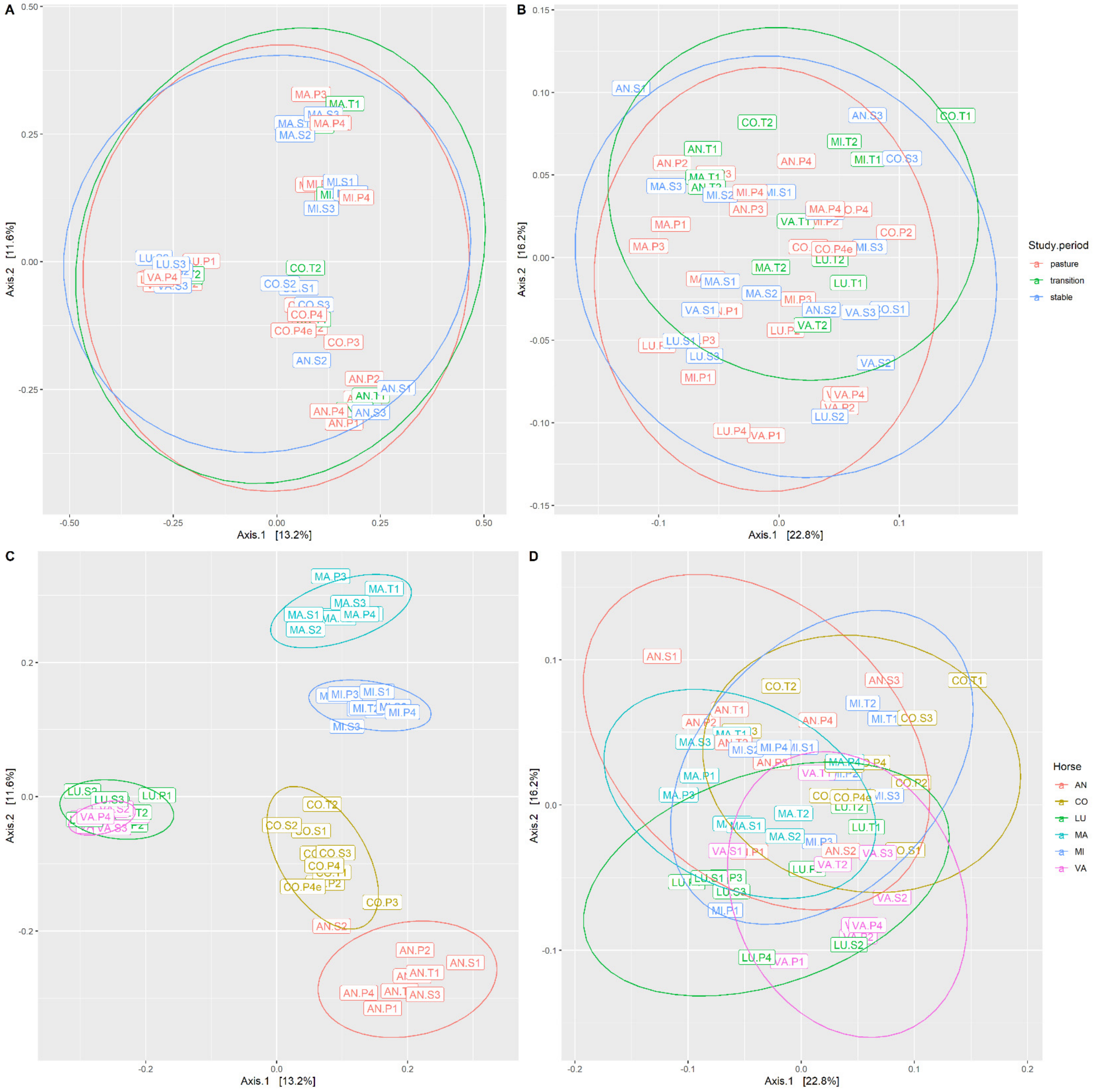

Figure S4. MDS of fecal samples using (a-b) Bray Curtis distance and (c-d) Weighted UniFrac distance. Points and ellipses colored by (a, b) study period, and (c, d) horse. Ellipses represent a 95% confidence level based upon multivariate t-distribution.

Table S3. Results of post hoc pairwise PERMANOVA for Bray Curtis distance between gastric microbiota of individual horses (AN, CO, LU, MA, MI, VA).

Contrasts	p value	FDR
ANxCO	0.215	0.269
ANxLU	0.173	0.266
ANxMA	0.040	0.120
ANxMI	0.022	0.098
ANxVA	0.132	0.248
COxLU	0.131	0.248
COxMA	0.250	0.288
COxMI	0.177	0.266
COxVA	0.330	0.330
LUxMA	0.025	0.098
LUxMI	0.026	0.098
LUxVA	0.205	0.269
MAxMI	0.271	0.290
MAxVA	0.065	0.163
MIxVA	0.016	0.098

* indicates statistical significance, FDR < 0.05

Table S4. Results of post hoc pairwise PERMANOVA for Bray Curtis distance between fecal microbiota of individual horses (AN, CO, LU, MA, MI, VA).

Contrasts	p value	FDR
ANxCO	0.001	0.001 *
ANxLU	0.002	0.002 *
ANxMA	0.001	0.001 *
ANxMI	0.001	0.001 *
ANxVA	0.001	0.001 *
COxLU	0.001	0.001 *
COxMA	0.001	0.001 *
COxMI	0.001	0.001 *
COxVA	0.001	0.001 *
LUxMA	0.001	0.001 *
LUxMI	0.001	0.001 *
LUxVA	0.001	0.001 *
MAxMI	0.001	0.001 *
MAxVA	0.001	0.001 *
MIxVA	0.001	0.001 *

* indicates statistical significance, FDR < 0.05.

Table S5. Results of two-way PERMANOVA, a phylogenetic distance-based method to test association between study week, horse, and week:horse interaction on Bray Curtis distance within each sample type and housing location.

Housing location	Sample type	Model terms	Sum Squares	Df	P value
Pasture	Gastric	Week	0.0742	1	0.9
		Horse	1.72	5	0.02 *
		Week:Horse	0.664	5	0.8
		Residuals	7.92	43	NA
	Fecal	Week	0.232	1	0.09
		Horse	5.88	5	0.001 *
		Week:Horse	0.877	5	0.3
		Residuals	6.83	43	NA
Stable	Gastric	Week	0.0742	1	0.9
		Horse	1.72	5	0.02 *
		Week:Horse	0.664	5	0.9
		Residuals	7.92	43	NA
	Fecal	Week	0.232	1	0.09
		Horse	5.88	5	0.001 *
		Week:Horse	0.877	5	0.3
		Residuals	7.22	43	NA

Df = model term degress of freedom, Location = housing location (pasture or stable), * indicates $p < 0.05$.

Table S6. Results of differential abundance analysis for gastric fluid microbiota between housing locations (location) and study weeks (timepoint). Base mean = average normalized count values across all samples.

Phylum	Family	Species †	Base mean	Adjusted p-value	
				Study Period	Timepoint
Actinobacteriota	*Bifidobacteriaceae*	*Alloscardovia sp.*	7.51	0.0064	0.146
	Microbacteriaceae	*Curtobacterium sp.*	33.16	0.0259	0.031
		Frigoribacterium sp.	7.67	0.0480	0.346
Bacteroidota	*Prevotellaceae*	*Prevotella sp. (37)*	58.02	<0.001 *	<0.001 *
Firmicutes	*Aerococcaceae*	*Aerococcus sp.*	21.40	0.0212	0.146
	Carnobacteriaceae	*Unclassified sp.*	6.46	0.0026	0.600
	Clostridiaceae	*Clostridium sensu stricto 1 sp.*	59.15	<0.001 *	0.146
	Lachnospiraceae	*Unclassified sp. (10)*	33.35	<0.001 *	0.002
	Leuconostocaceae	*Weissella sp.*	28.05	0.0259	0.105
	Staphylococcaceae	*Staphylococcus sp. (2)*	39.09	<0.001 *	0.146
	Streptococcaceae	*Streptococcus sp. (47)*	403.20	<0.001 *	0.146
		Streptococcus sp. (43)	989.26	0.0064	>0.9
		Streptococcus sp. (44)	227.66	<0.001 *	0.125
		Streptococcus sp. (45)	280.14	<0.001 *	>0.9
		Streptococcus sp. (37)	13.01	<0.001 *	0.312
		Streptococcus sp. (22)	81.10	<0.001 *	>0.9
		Streptococcus sp. (30)	13.12	0.0011	0.056
		Streptococcus sp. (40)	4.91	0.0015	0.184
		Streptococcus sp. (28)	6.77	0.0049	0.423
		Streptococcus sp. (4)	48.77	0.0111	0.346
		Streptococcus sp. (29)	3.44	0.0259	0.836
	Veillonellaceae	*Veillonella sp. (7)*	103.85	<0.001 *	0.001
Fusobacteriota	*Leptotrichiaceae*	*Leptotrichia sp. (3)*	41.67	<0.001 *	0.047
Proteobacteria	*Beijerinckiaceae*	*Methylobacterium-Methylorubrum adhaesivum (2)*	26.50	<0.001 *	0.028
		Methylobacterium-Methylorubrum sp. (3)	10.57	<0.001 *	0.012
		Methylobacterium-Methylorubrum sp. (4)	5.79	0.0024	0.801
	Comamonadaceae	*Variovorax sp. (1)*	18.09	<0.001 *	0.223
	Enterobacteriaceae	*Escherichia-Shigella sp.*	25.40	0.0071	>0.9
	Erwiniaceae	*Pantoea sp. (2)*	30.48	<0.001 *	0.001
		Pantoea sp. (1)	28.00	0.0011	0.015
	Moraxellaceae	*Moraxella sp. (3)*	6.22	0.0320	>0.9
	Neisseriaceae	*Alysiella sp. (1)*	76.20	<0.001 *	<0.001 *
		Neisseria sp.	5.29	0.0259	>0.9
	Pasteurellaceae	*Actinobacillus minor (2)*	10.64	0.0059	0.056
		Actinobacillus equuli (4)	23.26	0.0168	>0.9
		Unclassified (33)	490.18	<0.001 *	>0.9
		Unclassified sp. (24)	9.59	0.0024	0.028
	Pseudomonadaceae	*Pseudomonas sp. (8)*	24.99	0.0389	0.146
	Rhizobiaceae	*Allorhizobium-Neorhizobium-Pararhizobium-Rhizobium sp. (4)*	14.24	0.0259	0.138
	Sphingomonadaceae	*Sphingomonas sp. (6)*	5.65	<0.001 *	0.218
		Sphingomonas faeni (2)	21.51	0.0066	0.028
	Xanthomonadaceae	*Xanthomonas sp.*	13.43	0.0098	0.138

* indicates statistical significance, adjusted p-value < 0.05. † sp. indicates taxa was only classified to the genus level, taxa label number in ( ) if relevant

Table S7. Results of differential abundance analysis for fecal microbiota between housing locations (location) and study weeks (timepoint). Base mean = average normalized count values across all samples.

Phylum	Family	Species †	Base mean	Adjusted p-value	
				Location	Timepoint
Bacteroidota	*Bacteroidales BS11 gut group*	*Unclassified (8)*	6.89	0.008	>0.9
	Bacteroidales UCG-001	*Unclassified (7)*	10.54	0.005	>0.9
		Unclassified (10)	7.21	0.005	>0.9
	F082	*Unclassified (11)*	14.62	0.008	>0.9
	gir-aah93h0	*Unclassified*	69.74	0.014	>0.9
	Prevotellaceae	*Prevotellaceae UCG-001 sp. (2)*	12.82	<0.001 *	>0.9
		Prevotella sp. (50)	71.80	0.015	>0.9
		Prevotellaceae UCG-003 sp. (9)	18.84	0.020	>0.9
	Rikenellaceae	*Rikenellaceae RC9 gut group sp. (144)*	39.63	<0.001 *	0.030
		Rikenellaceae RC9 gut group sp. (52)	22.76	0.002	0.268
Firmicutes	*Anaerovoracaceae*	*[Eubacterium] nodatum group sp.*	8.23	<0.001 *	0.064
		Mogibacterium sp. (5)	6.64	0.029	0.669
	Butyricicoccaceae	*UCG-008 sp. (4)*	11.11	0.006	>0.9
	Erysipelatoclostridiaceae	*UCG-004 sp. (50)*	2.67	0.047	>0.9
	Erysipelotrichaceae	*Catenisphaera sp. (3)*	2.92	0.021	>0.9
	Lachnospiraceae	*Unclassified (6)*	9.15	<0.001 *	>0.9
		Lachnospiraceae AC2044 group sp. (49)	7.02	0.002	>0.9
		Unclassified (177)	17.70	0.002	>0.9
		Unclassified (99)	14.83	0.029	0.200
		Lachnospiraceae XPB1014 group sp. (58)	8.77	0.037	>0.9
		Cellulosilyticum sp. (3)	3.54	0.047	>0.9
		Lactobacillus hayakitensis	60.74	<0.001 *	0.007
		Lactobacillus equigenerosi	17.80	<0.001 *	0.012
	Leuconostocaceae	*Weissella sp.*	10.90	0.001	>0.9
	Oscillospiraceae	*Colidextribacter sp. (4)*	8.74	<0.001 *	>0.9
		NK4A214 group sp. (104)	7.54	0.047	>0.9
	Ruminococcaceae	*Ruminococcus sp. (11)*	22.43	0.024	>0.9
		Ruminococcus flavefaciens (1)	4.40	0.047	>0.9
	Streptococcaceae	*Streptococcus sp. (13)*	209.65	<0.001 *	0.012
	UCG-010	*Unclassified (51)*	9.19	0.015	>0.9
Proteobacteria	*Enterobacteriaceae*	*Escherichia-Shigella sp.*	11.61	0.001	>0.9
Spirochaetota	*Spirochaetaceae*	*Treponema sp. (79)*	6.35	<0.001 *	0.015
		Treponema sp. (106)	20.68	<0.001 *	>0.9
		Treponema sp. (100)	30.82	0.024	>0.9
		Treponema saccharophilum (4)	23.13	0.029	0.403
		Treponema sp. (54)	6.80	0.047	>0.9
Verrucomicrobiota	*Unclassified*	*Unclassified (990)*	25.39	0.005	>0.9
		Unclassified (743)	9.31	0.020	>0.9

* indicates statistical significance, adjusted p-value < 0.05. † sp. indicates taxa was only classified to the genus level, Unclassified indicates taxa not classified at genus level, taxa label number in ( ) if relevant
